# Correspondence

**Published:** 1823-03-01

**Authors:** 


### INTELLIGENCE, CORRESPONDENCE, &c.

From the following intelligence in the 20th number of the AMERICAN MEDICAL RECORDER, it will be seen that the MEDICO-CHIRURGICAL REVIEW has, ere this, become a Denizen of the United States.

" J. V. Seaman, New-York, has in Press, and will publish in Quarterly Numbers, *The Medico-Chirurgical Review*, and *Journal of Medical Science*;—conducted by associated Physicians and Surgeons, and superintended by JAMES JOHNSON, M.D. of the Royal College of Physicians, London.

" Each number of this work will contain upwards of 230 pages, forming a volume of nearly 1000 pages a year; each volume will contain three engravings. The price will be 5 dollars per annum, payable on the delivery of the first number.

" There are eleven numbers of this Journal now published in London, and it is proposed to commence at the first number, and to continue to reprint one number every month or six weeks, until we come up with the last number published in London, after which a number will be published regularly once a *Quarter*, in less than two months after its appearance in London.

" *** *Subscriptions for this valuable work will be received at the office of the American Medical Recorder.*"

While this event occasions a considerable pecuniary loss to the Editor,* he has some reason to be proud that his work is the first, and, he believes, the only journal of medical science, that has ever before received the honour of transplantation from the continent of Europe to the continent of America, or indeed to any foreign soil.

We are not without hopes that our labours may conduce to harmonize the profession on both sides of the Atlantic, and diffuse " peace and good will," as well as professional information, throughout the various regions which this Journal is now destined to travel. It must be some gratification to authors to know that the analyses of their works take now an extensive circuit in this Review on both sides of the Atlantic.

We have received a letter from Dr. J. M. Good, explaining the error which his printer made by converting *lb.*28 into *six hundred and twenty-eight*, mistaking the *b* for a 6, and thus raising the weight of a diseased

* The American demand will now be supplied in their own market.

liver to the above monstrous quantity.—See page 595. The leaf containing this error was early cancelled, but the notice did not reach us in time.

---

The long letter of PHILO-CRITICUS has reached us, but all his rhetoric will not persuade us to deviate from dense analysis and sparing criticism. Let him recollect that to veil oneself with an anonymous mask, and then strike unseen at every passenger, is not bravery and independence, but cowardice and assassination. Those hypercritics who call out most loudly for rigid severity on the writings of others, are, *to our knowledge*, abortive authors and unsuccessful journalists themselves. No man is so apt to impute improper *motives* to others, as those who are under the influence of improper motives themselves; and if we ever shall condescend to unmask these anonymous critics, the *motives* of *their* praises and censures will stand conspicuous before their brethren, and render themselves very low indeed in the eyes of the world.—*Verbum Sat.*

---

### CASE OF MR. KNOX.

The Medical Intelligencer says, "Perhaps Dr. Johnson may recollect that one (of the physicians) pointed out to him the fact at his visit, *and traced the edge of the liver for his information.*" Dr. Johnson is under the necessity of denying this statement. The condition of Mr. Knox's abdomen, from blisters, totally precluded any such demonstration, and Mr. Knox's words to Dr. Johnson, in the presence of Dr. Armstrong, were these:—"Two eminent anatomists have declared to me that there is no enlargement of my liver." Dr. Armstrong has, since the publication of the case, drawn Dr. Johnson's attention to a private conference which they had together the first night of meeting at Mr. Knox's house, in which Dr. A. stated his belief that Mr. Knox's liver was enlarged, and that he thought he could trace the edge of it in the abdomen. Dr. Johnson has some recollection of this observation, though it did not recur to him at the time the notes were made of the case. The object of the publication of the case was to shew that two eminent anatomists had been unable to detect any enlargement of the diseased viscus; and as Dr. J. made no examination, and gave no opinion himself, he could not possibly have any object in exaggerating or lessening any circumstance connected with it. Dr. Johnson could not but feel surprised at the *manner* in which the Medical Intelligencer displayed, or rather distorted the observation of Dr. Armstrong above alluded to, (which was totally unimportant in the narrative,) very clearly indicating a wish to attach a stigma on Dr. Johnson's veracity. Dr. J. would advise the Medical Intelligencer not to be too hasty in imputing evil to its neighbours.

---

Mr. Hutchinson's letter contains satisfactory reasons why, in the second edition of his work, he was sparing of his own cases, and anxious to bring forward the testimonies of others.

---

We beg to direct the attention of our surgical brethren to the series of engravings of newly invented or improved surgical instruments, now publishing in this Journal by Mr. Weiss.

---

### HUNTERIAN SOCIETY.

On Wednesday, February the 5th, the anniversary of this Society was held at the Society's Room, Aldermanbury, when the following members were elected officers for the ensuing year:—

*President*, Benjamin Robinson, M. D. *Vice Presidents*, William Babington, M. D. F. R. S. H. Lidderdale, M.D. Sir William Blizard, F.R.S. Benjamin Travers, Esq. F. R. S. *Treasurer*, B. Robinson, M. D. *Secretaries*, J. T. Conquest, M. D. F. L S. William Cooke, Esq. *Council*, Thomas Callaway, Esq. W. D. Cordell, Esq. John Dunston,

Esq. F.L.S. H. Greenwood, Esq. John Winstone, Esq. H. Hawkins, Esq. J. C. Knight, Esq. Lewis Leese, Esq. Eusebius A. Lloyd, Esq. J. Miles, Esq. B. C. Pierce, M. D. J. Roberts, Esq.

On the following day the members and friends of the Society dined together at the London Tavern—on which occasion Dr. Robinson took the chair. The report of the progress of the Society, and of the state of its finances, is encouraging.

